# LM2KM: feature extraction from pretrained protein and molecular language models combined with quantum mechanics (QM) features for *K*_M_ predictions

**DOI:** 10.1039/d6dd00139d

**Published:** 2026-10-20

**Authors:** Jingchen Zhai, Lianjin Cai, Taoyu Niu, Lei Xie, Junmei Wang

**Affiliations:** a Department of Pharmaceutical Sciences and Computational Chemical Genomics Screening Center, School of Pharmacy, University of Pittsburgh Pittsburgh Pennsylvania 15261 USA juw79@pitt.edu; b Center for Drug Discovery & School of Pharmacy and Pharmaceutical Sciences, Northeastern University Boston MA USA; c Department of Computer Science, Hunter College, The City University of New York New York NY USA lxie@iscb.org

## Abstract

The Michaelis constant (*K*_M_) is a key parameter for characterizing enzyme-substrate interactions and plays an essential role in enzyme kinetics modeling and physiologically based pharmacokinetic (PBPK) applications. However, experimental determination of *K*_M_ is labor-intensive, and existing computational prediction approaches remain limited in accuracy and scalability. In this study, we systematically evaluated pretrained protein and molecule language models for *K*_M_ prediction by integrating four protein language models (PLMs) and two molecule language models (MLMs) to encode enzyme sequences and substrate structures, respectively. Principal component analysis (PCA) was applied to reduce feature dimensionality, and a wide range of representation combinations were assessed. Our results indicated that protein representations derived from the last four layers of ProtT5 using approximately 70 features, combined with 20 CLS-based molecular features from MolFormer, provided a well-balanced and efficient representation for *K*_M_ prediction. In addition, the incorporation of quantum mechanics (QM) descriptors consistently improved prediction performance across all tested configurations, with the model *R*^2^ of 0.537. In summary, this work provides a flexible and extensible framework for large-scale *K*_M_ prediction, with strong potential to facilitate enzyme pathway identification, drug and lead screening, and sequence-based protein design, supporting broader applications in enzyme kinetics-driven modeling and drug development.

## Abstract 2

The Michaelis constant (*K*_M_) is a key parameter for characterizing enzyme-substrate interactions and plays an essential role in enzyme kinetics modeling and physiologically based pharmacokinetic (PBPK) applications.

## Graphical abstract

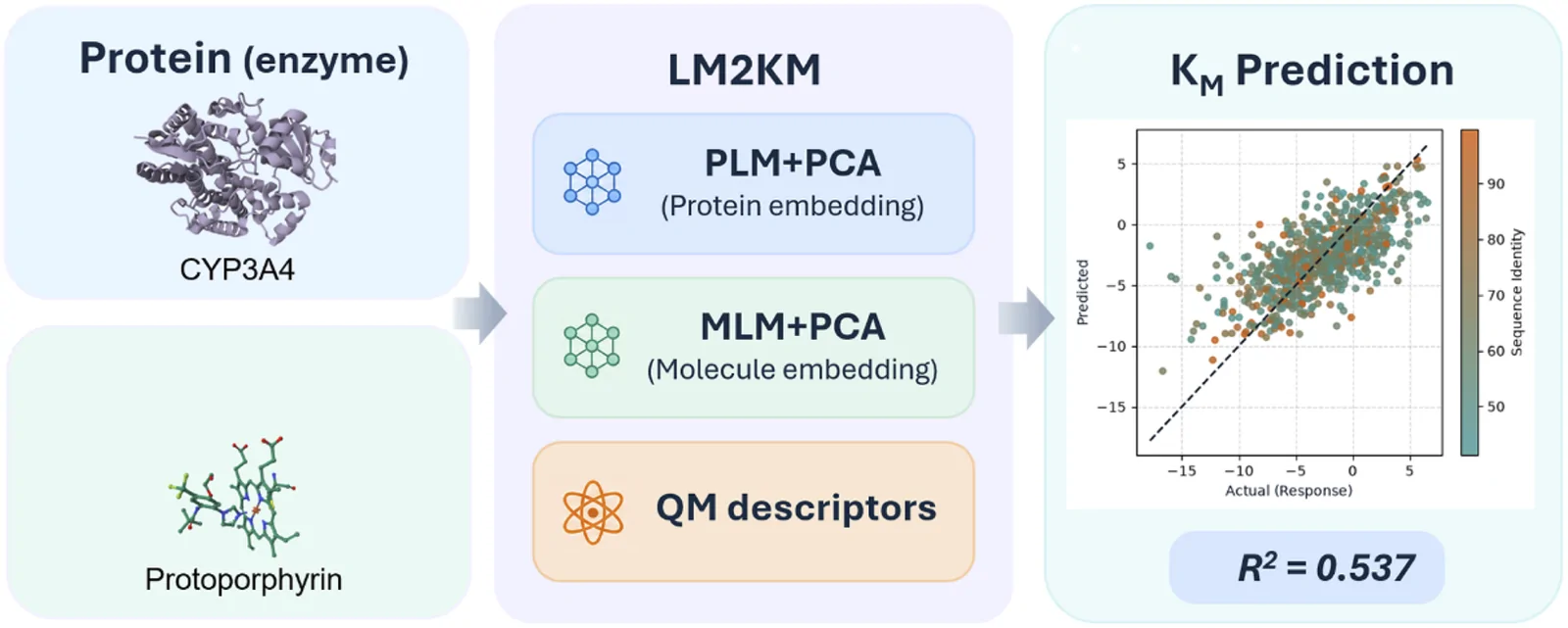

## Introduction

Understanding drug pharmacokinetic (PK) processes is a key part in drug development, among which metabolic clearance remains one of the most challenging components to characterize.^1,2^ Physiologically based pharmacokinetic (PBPK) modeling, as a bottom-up approach, offers several advantages over traditional top-down population PK modeling, including reduced risk of overfitting and a stronger reliance on mechanistic descriptions of biological processes.^3–5^ Within PBPK models, enzyme kinetics parameters play a critical role in describing drug metabolism.^6^ In particular, the Michaelis constant (*K*_M_) represents the substrate concentration at which the reaction rate reaches half of its maximum value and serves as a key determinant of enzyme-substrate affinity.^7^ However, experimental measurement of *K*_M_ is labor-intensive and often unavailable, especially for compounds in early drug discovery stages.^8^ The prediction of *K*_M_ remains highly challenging, with limited available tools and suboptimal predictive performance, resulting in substantial data gaps that hinder PBPK model construction for new molecular entities (NMEs).^9^

Prior to the wide application of machine learning, physics-based approaches such as quantum mechanics (QM) and molecular mechanics (MM) methods were applied to *K*_M_ prediction.^10,11^ These methods estimate activation free energies on QM/MM potential energy surfaces and subsequently derive apparent *K*_M_ through transition state theory or related kinetic frameworks. In parallel, quantitative structure–activity relationship (QSAR) models were widely used to predict *K*_M_ by relating computable molecular structural and physicochemical descriptors of substrates to experimental measurements using regression or other statistical techniques, thereby enabling extrapolation to new compounds.^12,13^

With recent advances in machine learning and deep learning, data-driven approaches, including deep neural networks and graph neural networks, have been increasingly applied to predict enzyme kinetic parameters by learning molecular representations directly from data.^14–18^ More recently, pretrained protein and molecule language models are increasingly applied for feature extraction, further facilitating such prediction tasks.^19,20^ Compared with the traditional physics-based methods, machine learning-based approaches enable greatly improved computational efficiency and are more suitable for large-scale screening applications.^21^ In particular, recent years have seen the rapid proliferation of numerous protein and molecule language models with similar underlying principles and architectures.^22^ However, systematic comparisons across different language model-based approaches remain challenging due to non-unified evaluation metrics and heterogeneous training datasets. Moreover, purely data-driven models often suffer from limited interpretability and generate high-dimensional representations that may be suboptimal for downstream prediction tasks. In addition, some existing machine learning studies involving protein sequences rely on enzyme commission (EC) numbers for data curation, overlooking the fact that a single protein sequence may correspond to multiple EC annotations, which can introduce ambiguity into model inputs and compromise data quality.^14^

In this work, we systematically integrated and leveraged four pretrained protein language models (PLMs) and two molecule language models (MLMs) to generate embeddings for *K*_M_ prediction, with the goal of identifying the most suitable language model combinations and feature dimensionalities for this task. This modeling effort was supported by a large and carefully curated dataset, which was constructed through extensive data cleaning and validation procedures to ensure high data quality. In addition to language model-derived representations, quantum mechanics (QM) features were incorporated to provide complementary descriptions of chemical reactivity, enabling a more comprehensive characterization of enzyme-substrate interactions beyond purely statistical representations.

Notably, compared with previous studies, data curation in this work was performed at the protein sequence level rather than based on EC numbers. Furthermore, our framework can process reactions involving multiple protein sequences. To address input length limitations inherent to many protein language models, an overlapped sliding-window strategy was applied, allowing effective representation of long protein sequences.

Our study provides a practical framework for directly predicting *K*_M_ values from protein sequences and molecular representations, without requiring experimentally determined three-dimensional protein and molecule structures. This approach enables the identification of potential metabolic pathways, the screening of candidate substrates for specific enzymes, and the estimation of relevant pharmacokinetics parameters, thereby supporting PBPK model development under limited data availability. In addition, the proposed framework has the potential to be extended toward protein sequence engineering by linking sequence-level variations to functional kinetic outcomes. Finally, this work demonstrates the power of unifying machine learning representations with physics-based descriptors, motivating continued advances at the interface of statistical learning and mechanistically grounded chemical modeling. Together, this study establishes a sequence-based and mechanism-informed framework for *K*_M_ prediction that integrates modern representation learning with physicochemical insights, providing a scalable solution to support PBPK modeling and related applications.

## Result

### Overview of the modeling framework and dataset

In this work, we presented a large language model-based framework, LM2KM (Language Model to *K*_M_) for *K*_M_ prediction. The LM2KM modeling framework is summarized in Fig. 1. Protein amino acid sequences and substrate Simplified Molecular Input Line Entry System (SMILES) strings were used as inputs to the corresponding PLMs and MLMs to extract protein and molecule embeddings. For protein sequences exceeding the input length limitation of the language models, an overlapped sliding-window strategy was applied, as illustrated in Fig. 2. Principal component analysis (PCA) was subsequently performed on the embeddings generated by each language model, and principal components (PCs) were ranked according to their explained variance. Different numbers of top-ranked PCs, together with different PLM–MLM combinations, were systematically evaluated to identify configurations that yield optimal *K*_M_ prediction performance. Detailed methodological descriptions are provided in the Methods section.

**Fig. 1:**
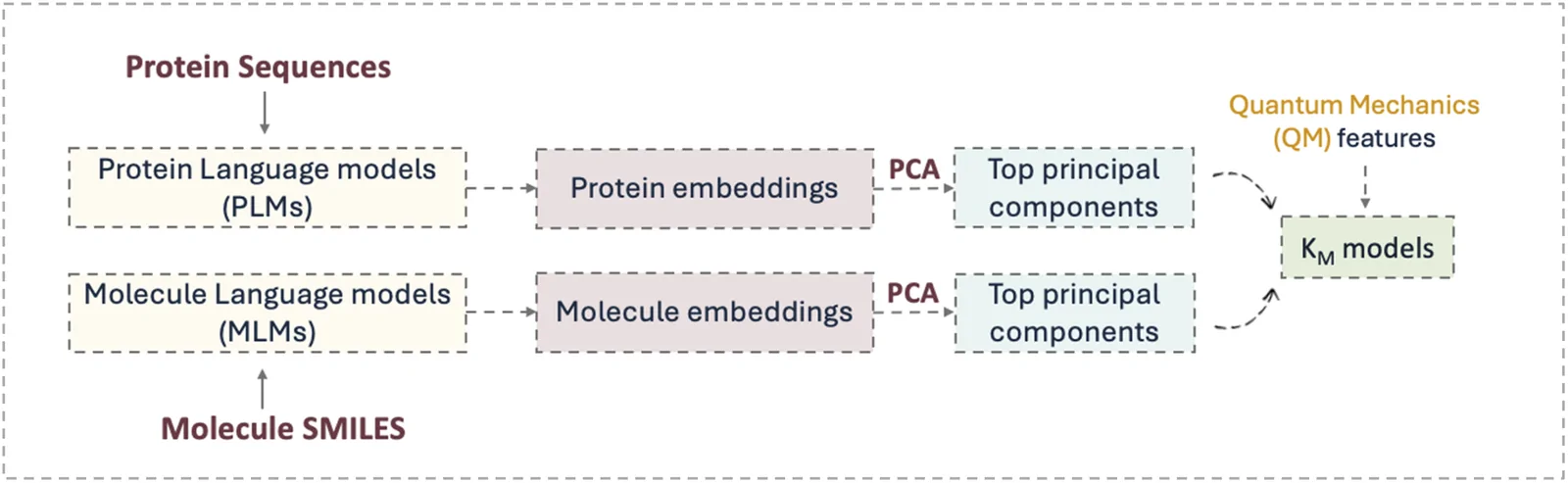
Overview of the LM2KM modeling framework.

**Fig. 2:**
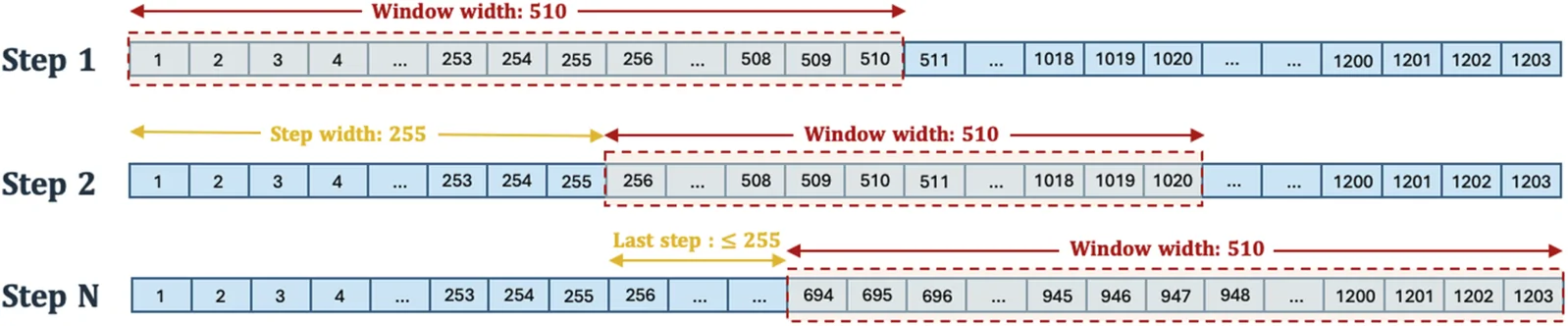
Overlapped sliding-window strategy for protein sequence embedding extraction. For protein sequences exceeding the input length limit of the language models, an overlapped sliding-window approach is applied. Each window includes 510 amino acids with a step size of 256 amino acids, resulting in partial overlap between adjacent windows to preserve local contextual information. Sliding windows are generated sequentially along the full sequence, with the final step adjusted to ensure complete coverage of the input sequence.

The curated dataset used in this study comprises a total of 20 462 *K*_M_ records (Fig. 3). Most substrate SMILES strings have lengths shorter than 200 characters, and most protein sequences are shorter than 2000 amino acids. Among all records, 18 225 reactions involve a single protein, 1556 reactions involve two proteins, and the remaining records involve more complex multi-protein systems. In terms of enzyme classification, 7103 reactions belong to EC category 1 (oxidoreductases), representing slightly more than one-third of the dataset and constituting the largest enzyme class. This is followed by EC categories 2 (transferases) and 3 (hydrolases), which together account for approximately half of the dataset. The detailed distribution of enzyme classes is shown in Fig. 3D.

**Fig. 3:**
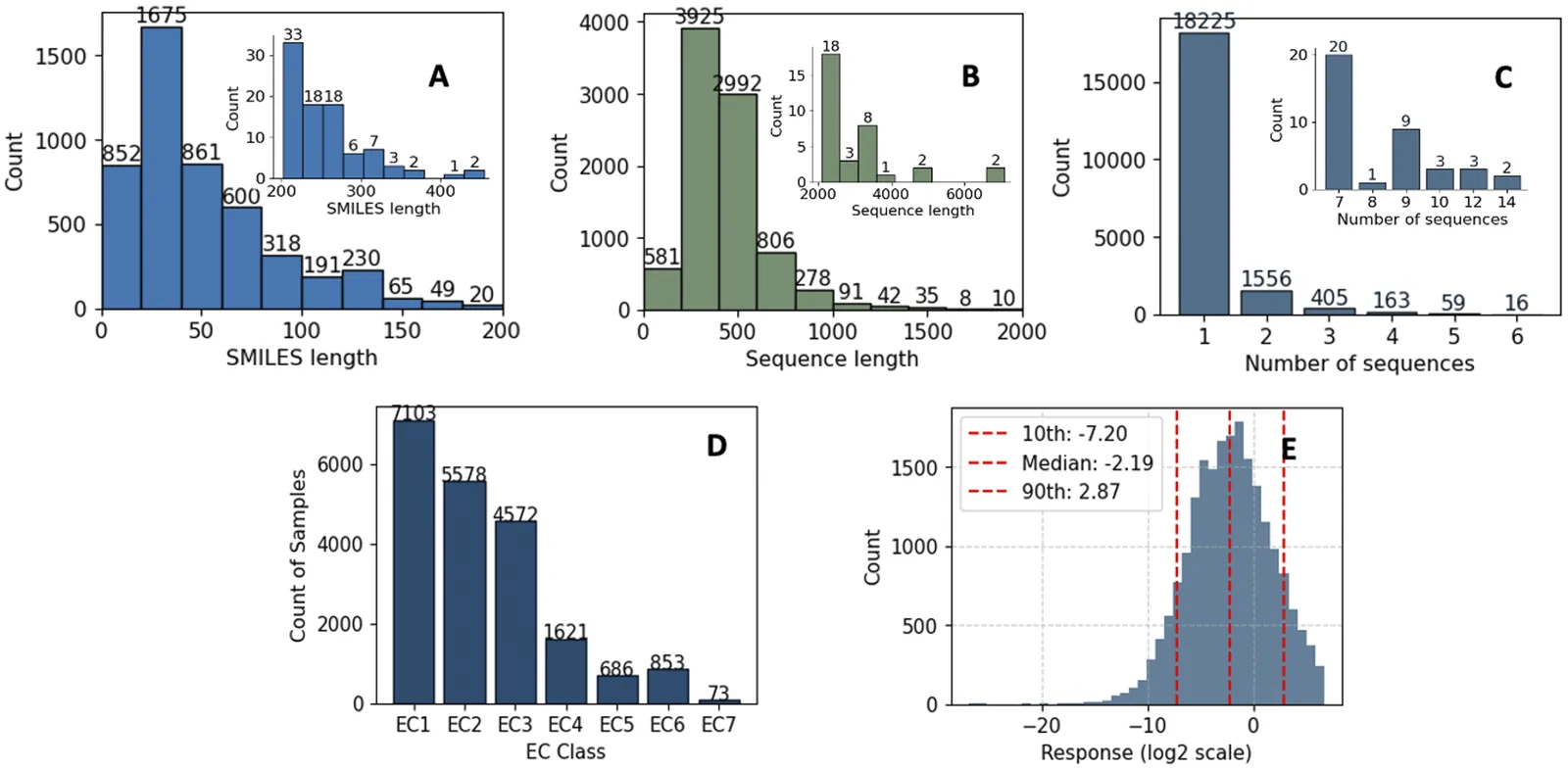
Distribution and composition of the *K*_M_ dataset. (A) Distribution of molecular SMILES string lengths; (B) distribution of individual protein sequence lengths; (C) number of proteins associated with each *K*_M_ record; (D) number of metabolic reactions in each of the seven Enzyme Commission (EC) classes; and (E) distribution of log_2_-transformed *K*_M_ values across all records.

After log_2_ transformation, the *K*_M_ values exhibit a near-normal distribution (Fig. 3E), with a median value of −2.19. The 10th and 90th percentiles are −7.20 and 2.87, respectively, indicating a broad dynamic range of enzyme-substrate affinities represented in the dataset.

Protein and molecule embedding extraction from PLMs and MLMs and PCA transformation.

In total, ten protein-derived feature representations obtained from PLM embeddings and four molecule-derived feature representations obtained from MLM embeddings were constructed for downstream *K*_M_ prediction (Table 1). Detailed procedures for embedding extraction are described in the Methods section. The convergence behavior of PCA for each feature set is summarized in Fig. 4.

**Table 1:** Feature representations derived from PLMs and MLMs. *If a sliding-window strategy is applied, CLS and pooled embeddings are obtained by averaging the corresponding outputs across all sliding windows

Feature name	Description of embedding extraction strategy prior to PCA
protBERT_mean	Mean-pooled amino-acid embeddings from the protBERT model
protBERT_cls	Classification-token (CLS) embedding from the protBERT model*
protBERT_pooler	Pooled embeddings from the classification token of the protBERT model *
ESM2_mean	Mean-pooled amino-acid embeddings from the esm2 model
ESM2_cls	Classification-token (CLS) embedding from the esm2 model*
ESM2_pooler	Pooled embeddings from the classification token of the esm2 model*
ProtT5_last1	Mean-pooled amino acid-level embeddings from the final layer of the ProtT5 model
ProtT5_last4	Mean-pooled amino acid-level embeddings averaged across the last four layers of the ProtT5 model
Ankh_last1	Mean-pooled amino acid-level embeddings from the final layer of the Ankh model
Ankh_last4	Mean-pooled amino acid-level embeddings averaged across the last four layers of the Ankh model
ChemBERTa_cls	Classification-token (CLS) embedding from the ChemBERTa model
ChemBERTa_pooler	Pooled embedding derived from the CLS-based representation of the ChemBERTa model
MolFormer_cls	Classification-token (CLS) embedding from the MolFormer model
MolFormer _pooler	Pooled embedding derived from the CLS-based representation of the MolFormer model

**Fig. 4:**
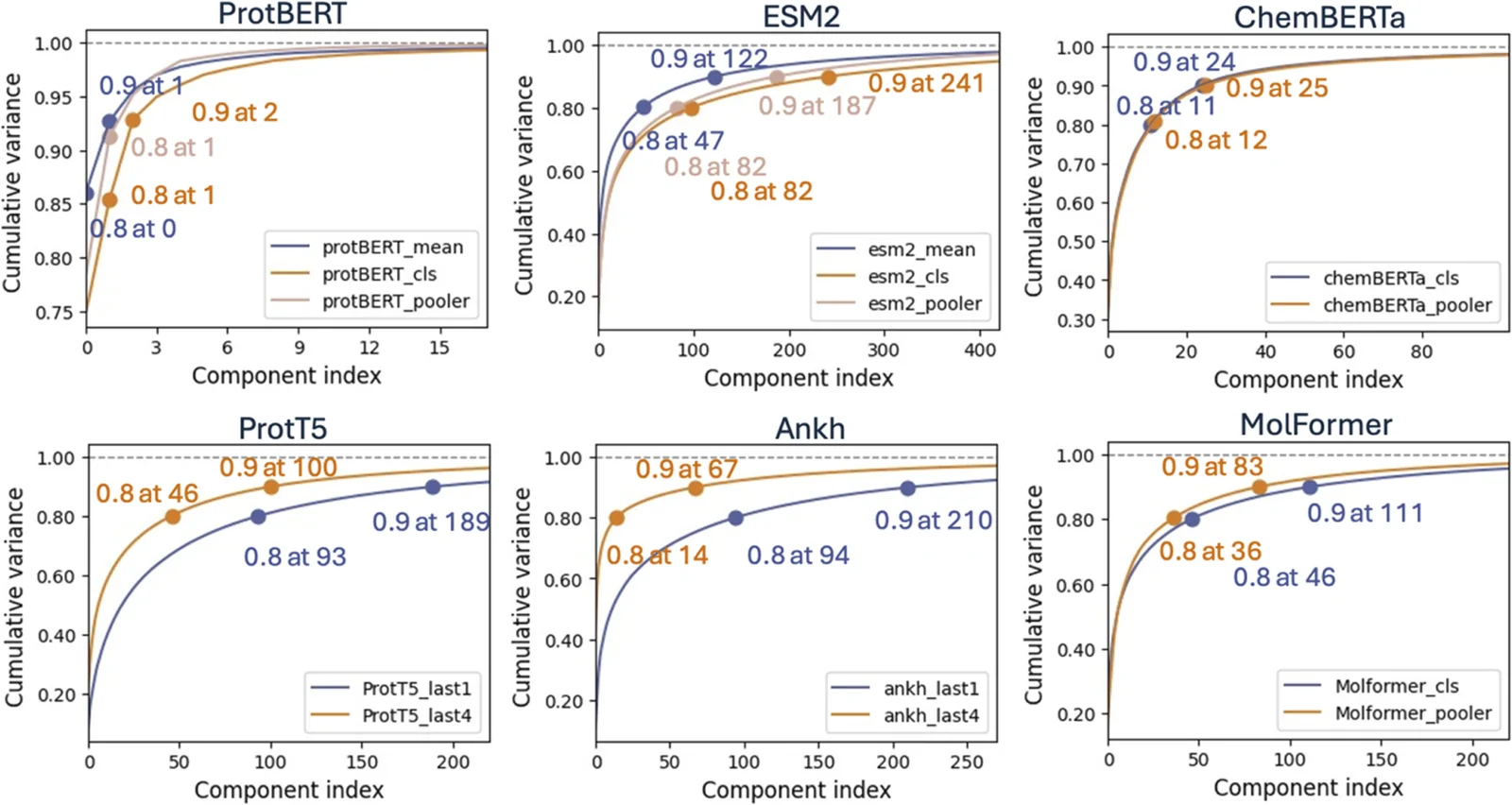
PCA convergence of PLM and MLM derived feature representations. Each panel shows the cumulative explained variance *versus* principal component rank for features derived from a given language model. Colored curves denote different feature extraction strategies from the same model (as indicated in the legend of each panel). The annotated labels (“0.8 at k” and “0.9 at k”) indicate the minimum number of top-ranked principal components (k) required to reach 80% and 90% cumulative explained variance, respectively.

Among protein-derived features, differences in PCA convergence behavior are observed across PLMs and extraction strategies. ProtBERT-derived features exhibit extremely rapid convergence, requiring only one to two principal components to explain 90% of the total variance, depending on the embedding extraction strategy. In contrast, features derived from ProtT5_last1, ESM2_cls, ESM2_pooler, and Ankh_last1 show slower convergence, with approximately 200 or more principal components required to reach 90% cumulative explained variance. Other PLM-based feature representations display intermediate behavior, typically requiring around 100 principal components to explain 90% of the variance. For molecule-derived features, ChemBERTa exhibits relatively fast convergence, reaching 90% cumulative explained variance with approximately 25 principal components. In comparison, MolFormer-derived features converge more slowly, requiring around 100 principal components to explain 90% of the variance.

Overall, these results demonstrate variation in the intrinsic dimensionality and redundancy of language model-derived features, indicating that different PLMs and MLMs encode biochemical information with varying degrees of compactness and suitability for downstream *K*_M_ prediction.

### Performance of different PLM–MLM combinations and effect of feature dimensionality on *K*_M_ prediction

We systematically combined PLMs and MLMs to construct *K*_M_ prediction models by exhaustively pairing one PLM with one MLM. For each language model, two feature dimensionalities were evaluated by retaining either the top 20 or top 60 PCs after PCA. At this step, PLM and MLM features with 20 PCs were only combined with each other, and likewise, 60-PC PLM features were exclusively paired with 60-PC MLM features. The performance of all individual model combinations is summarized in SI Fig S1 and S2.

To facilitate comparison across different feature representations, we calculated the average prediction performance for each feature by averaging the *R*^2^ values obtained from all corresponding PLM–MLM combinations. These aggregated results are shown in Fig. 5. Among protein-derived features, ProtT5_last4_60 achieved the highest overall performance at the 60-PC dimensionality (*R*^2^ = 0.503), whereas ProtT5_last4_20 performed best among protein features at the 20-PC dimensionality (*R*^2^ = 0.493). Similarly, for molecule-derived features, MolFormer_cls_60 (*R*^2^ = 0.489) and MolFormer_cls_20 (*R*^2^ = 0.476) consistently outperformed other MLM-based representations at the 60-PC and 20-PC dimensionalities, respectively.

**Fig. 5:**
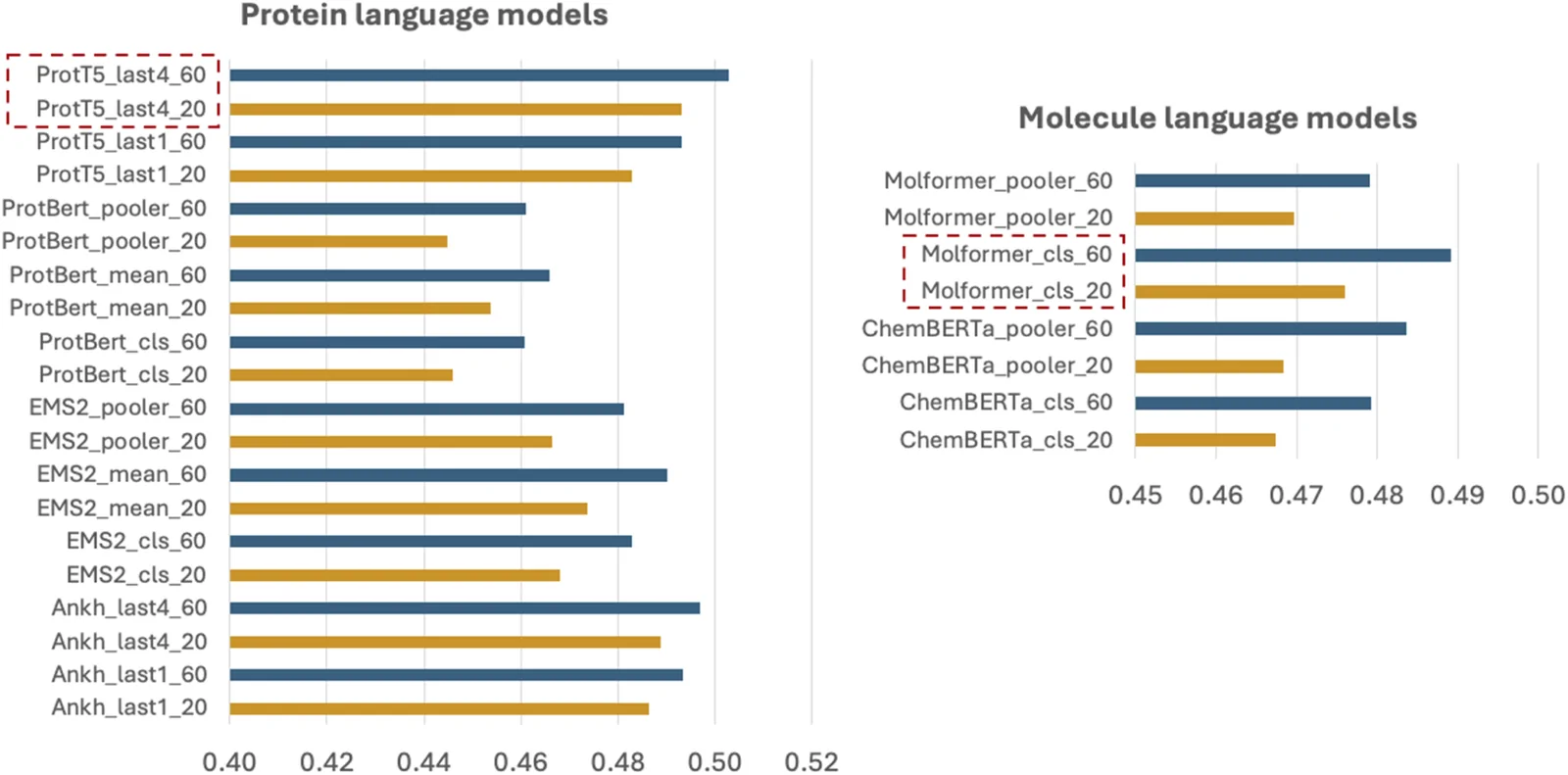
Average prediction performance of individual feature representations. The bar plot shows the average *R*^2^ for each PLM and MLM, calculated by averaging *R*^2^ across all corresponding PLM–MLM combinations evaluated at the same feature dimensionality (20 PCs, yellow; 60 PCs, blue).

Since the above analysis was restricted to symmetric feature dimensionalities (*i.e.*, 20–20 and 60–60 combinations), we further evaluated asymmetric combinations to assess whether asymmetric dimensionalities perform better for proteins and molecules. Specifically, combinations of MolFormer_cls_20 with ProtT5_last4_60 and MolFormer_cls_60 with ProtT5_last4_20 were evaluated. As shown in Fig. 6, the combination of molecule features with 20 PCs and protein features with 60 PCs yielded the best predictive performance, achieving the highest *R*^2^ and lowest RMSE among all tested configurations. These results indicate that molecular embeddings reach sufficient representational capacity at lower dimensionality, whereas protein embeddings benefit from higher-dimensional representations, highlighting distinct feature requirements for substrate and protein.

**Fig. 6:**
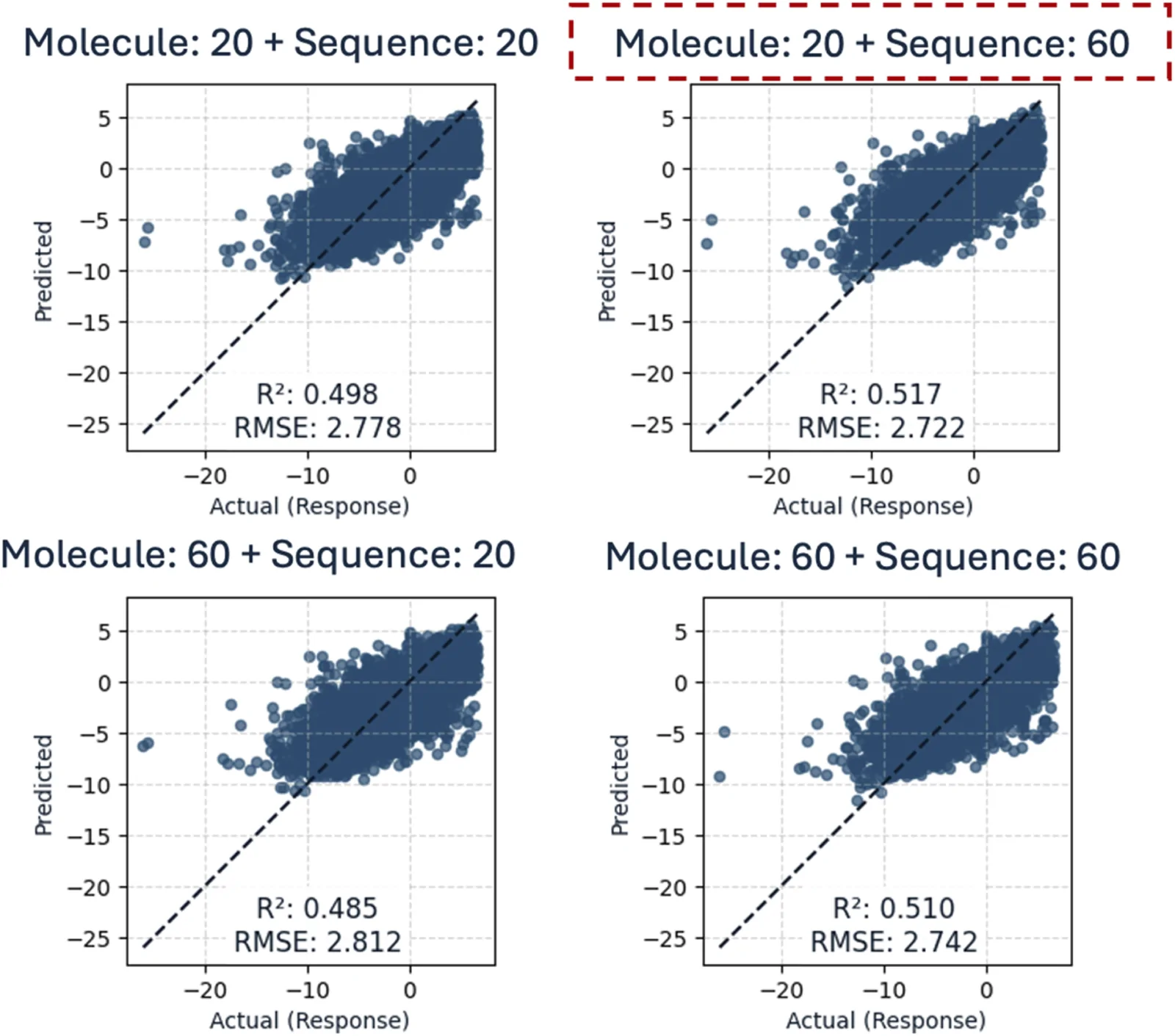
Prediction performance of ProtT5_last4 and MolFormer_cls feature combinations under different feature dimensionalities. The *x*-axis shows experimentally measured log_2_(*K*_m_) values, while the *y*-axis shows model-predicted log_2_(*K*_m_) values.

### Contribution of quantum mechanics (QM) features to model performance

Based on the above analyses, 20 molecule-derived features and 60 protein-derived features were identified as suitable for *K*_M_ model construction. To further explore whether additional physicochemical information could improve prediction performance, quantum mechanics (QM) features were incorporated, and the optimal feature dimensionality was further evaluated. For each molecule, geometry optimization was carried out at the ωB97XD/6-31G* level, followed by population analysis. The HOMO and LUMO energies were then used to derive global reactivity descriptors, including chemical potential, chemical hardness, chemical softness, electrophilicity index, electron-donating power, and electron-accepting power. A subset (*n* = 20 002) was constructed by removing molecules whose QM calculations were computationally prohibitive. The impact of QM-derived features on *K*_M_ prediction was then evaluated by training and testing models on this subset, with and without the inclusion of QM descriptors.

To assess the effects of feature dimensionality and QM descriptors on model performance, systematic comparisons were performed using identical training and test splits. We first evaluated feature combinations for the dataset without QM features. As shown in Fig. 7, increasing protein feature dimensionality from 60 to 70 features improved model performance, whereas further increasing to 80 features provided limited additional benefit. To further validate these observations, five additional parallel experiments were conducted using different random seeds for dataset splitting and model construction, and paired *t*-tests were performed to assess the statistical significance of performance differences among PCA component settings (Table 2). The results confirmed that increasing protein features from 60 to 70 components resulted in a significant improvement, while further increasing to 80 components did not provide a significant improvement compared with the 70-component setting. With protein features fixed at 70 components, varying molecular feature dimensionality by ±10 did not lead to significant performance improvements. Moreover, reducing the molecular representation from 20 to 10 PCs led to decreased performance in several experiments, suggesting that an insufficient molecular representation may compromise prediction accuracy. Based on these results, the combination of 70 protein-derived PCs and 20 molecule-derived PCs was selected as the optimal feature configuration for the subsequent analyses.

**Fig. 7:**
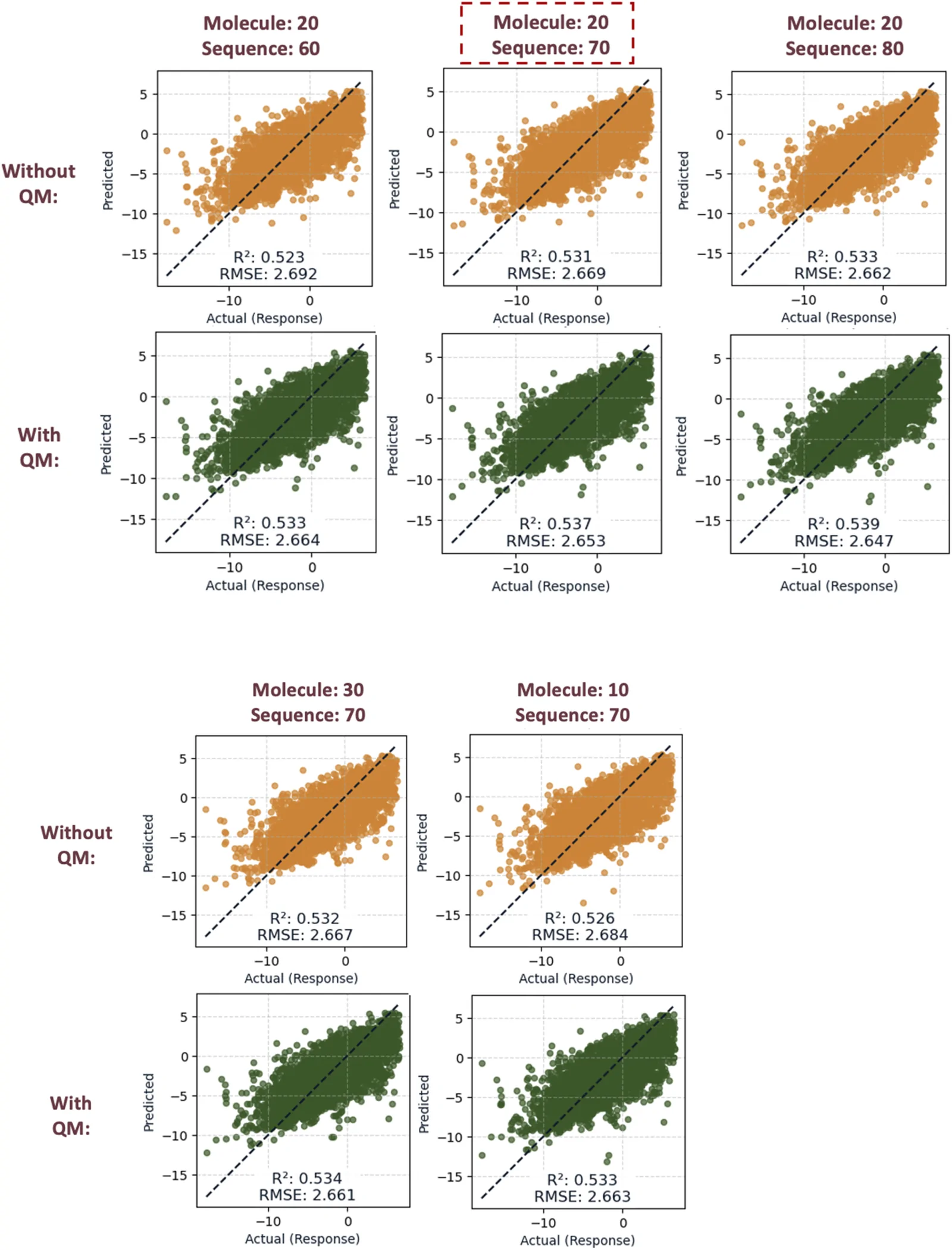
Effect of incorporating QM descriptors and feature dimensionality changing on *K*_M_ prediction performance. The results shown in the figure are from a set of representative experiments using an identical training and test split across different PCA configurations. The inclusion of QM descriptors consistently improved model performance across the five parallel runs, with a statistically significant increase in performance (two-sided paired *t*-test, *p* ≈ 0.008 < 0.01). The *x*-axis shows experimentally measured log_2_(*K*_m_) values, while the *y*-axis shows model-predicted log_2_(*K*_m_) values. All with-QM and without-QM comparisons were conducted using the same QM-available subset of 20 002 records.

Test set *R*^2^ values under different PCA settings across multiple parallel experiments

**Table:** 

	PCA component settings				
Different random settings	(A) Mol = 20, Seq = 60	(B) Mol = 20, Seq = 70	(C) Mol = 20, Seq = 80	(D) Mol = 30, Seq = 70	(E) Mol = 10, Seq = 70
Random setting 1 (Fig. 7)	0.523	0.531	0.533	0.532	0.526
Random setting 2	0.529	0.529	0.533	0.529	0.531
Random setting 3	0.530	0.532	0.538	0.529	0.529
Random setting 4	0.525	0.528	0.525	0.528	0.524
Random setting 5	0.517	0.520	0.523	0.520	0.519
Random setting 6	0.517	0.522	0.520	0.520	0.524
Random setting 7	0.520	0.521	0.518	0.519	0.523

**Table:** 

Comparison	*P*-value	Significant (*α* = 0.05)
A → B	0.0208	Yes
B → C	0.4960	No
B → D	0.1723	No
B → E	0.4198	No

Next, QM features were incorporated into PLM and MLM embeddings. Across all tested dimensionalities, models with QM descriptors consistently outperformed their counterparts without them (Fig. 7). According to a two-sided paired *t*-test across the five experiments, incorporating QM descriptors resulted in a statistically significant improvement in model performance across all evaluated PCA configurations (*p* = 0.0084). To further assess whether this improvement was dependent on a particular train-test partition, we performed an additional independent train-test split (Table 3). Consistent performance gains were observed after incorporating QM descriptors, and the improvement remained statistically significant across the evaluated PCA settings (*p* = 0.0077). These results demonstrate that the benefit of QM descriptors is robust and not attributable to a specific data split. This finding demonstrates that QM-derived descriptors provide complementary information to language model-based representations and consistently enhance *K*_M_ prediction performance.

**Table 3:** Robustness analysis of QM feature contributions across different train-test splits

	PCA component settings					
Different random settings	(A) Mol = 20, Seq = 60	(B) Mol = 20, Seq = 70	(C) Mol = 20, Seq = 80	(D) Mol = 30, Seq = 70	(E) Mol = 10, Seq = 70	Statistic significance
Random setting 1 (Fig. 7, without QM)	0.523	0.531	0.533	0.532	0.526	*p* = 0.0084
Random setting 1 (Fig. 7, with QM)	0.533	0.537	0.539	0.534	0.533	
Random setting 2 (without QM)	0.529	0.529	0.533	0.529	0.531	*p* = 0.0077
Random setting 2 (with QM)	0.533	0.537	0.538	0.532	0.534	

To further investigate the contribution of individual QM descriptors, we performed a one-at-a-time feature addition analysis in which each QM descriptor was individually added to the baseline model containing only PLM- and MLM-derived features. The analysis was repeated under three independent random settings to assess the robustness of the observations. As shown in Fig. 9, molecular hardness and softness consistently produced the largest individual improvements among the evaluated QM descriptors, and incorporating all QM descriptors resulted in a more robust improvement in predictive performance than adding individual descriptors alone. Although all the QM descriptors are derived from HOMO and LUMO through different nonlinear transformation, these pre-computed transformations can provide the model with more informative and interpretable combinations of the underlying electronic properties, rather than requiring the tree-based model to identify such relationships through numerical feature splits.

### Evaluation of model generalizability toward unseen molecules and protein sequences

To evaluate whether model performance was influenced by the presence of previously observed molecules or protein sequences in the training set, we further assessed prediction performance with respect to molecular and sequence novelty. As shown in Fig. 8A, samples containing molecules not observed during model training exhibited prediction behavior comparable to those containing molecules present in the training set, without apparent loss of predictive accuracy.

Next, we investigated the impact of protein sequence novelty on model performance. Test proteins whose sequences were not identical to any protein in the training set exhibited prediction accuracy comparable to that of proteins with identical sequences represented during training (Fig. 8B). To further examine the effect of sequence divergence, pairwise sequence identities between each test protein and its closest training-set homolog were calculated from MUSCLE multiple sequence alignments. No apparent correlation was observed between sequence identity and prediction performance. In particular, proteins with lower sequence identity to the training set did not exhibit larger prediction errors than those with higher sequence identity (Fig. 8C).

Together, these analyses suggest that the predictive performance of the model was not primarily driven by memorization of previously encountered molecules or highly similar protein sequences. Instead, the model retained predictive capability across a broad range of molecular structures and protein sequence identities, indicating its ability to generalize beyond the specific chemical and sequence space represented in the training set.

## Discussion

In this work, we systematically investigated the suitability of pretrained protein and molecule language model representations for *K*_M_ prediction. By integrating four PLMs for protein sequence encoding and two MLMs for substrate representation, we evaluated a wide range of embedding combinations and feature dimensionalities to identify configurations most appropriate for this task. We revealed that different PLMs and MLMs encode biochemical information in distinct ways, which may partly explain their varying effectiveness in downstream *K*_M_ prediction.

In benchmarking against current state-of-the-art studies, the most recent 2026 publication reported a top-performing *K*_M_ prediction model with *R*^2^ = 0.55 on the test set,^23^ slightly higher than our model (*R*^2^ = 0.537 for the model achieving the best balance between feature complexity and predictive performance, and *R*^2^ = 0.539 for the model with the highest overall predictive accuracy). Other published single-task *K*_M_ prediction models typically report *R*^2^ values around 0.50.^8^ However, direct comparison across different studies is inherently limited because of differences in datasets and training/test partitions.

**Fig. 8:**
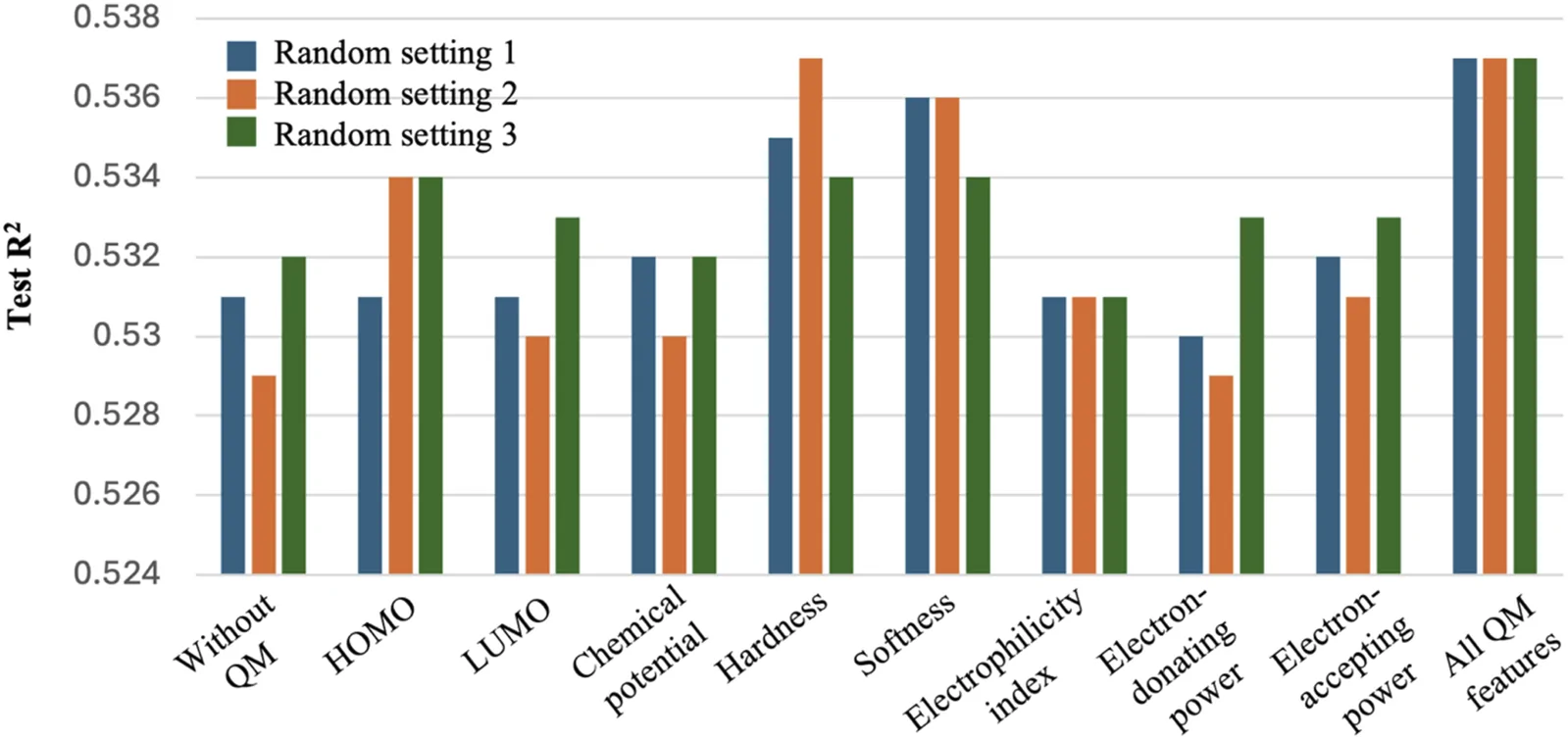
One-at-a-time feature addition analysis of QM descriptors across three independent random settings. The *y*-axis represents the respective test-set *R*^2^ value.

To enable a fair head-to-head comparison, we further evaluated LM2KM using the public Kroll benchmark dataset and the identical training/test split reported in the KmPred study. Under these matched experimental conditions, LM2KM achieved a test-set *R*^2^ of 0.509, comparable to the reported test performance of KmPred (*R*^2^ = 0.51), demonstrating that the proposed modeling framework performs competitively on a common public benchmark. Notably, this benchmark evaluation was conducted using only PLM- and MLM-derived descriptors. QM descriptors were not included because of the substantial computational cost associated with the considerably larger benchmark dataset. As QM descriptors consistently and significantly improved model performance in our primary experiments, the benchmark result presented here should be regarded as a conservative estimate of the performance capability of the complete LM2KM framework.

**Fig. 9:**
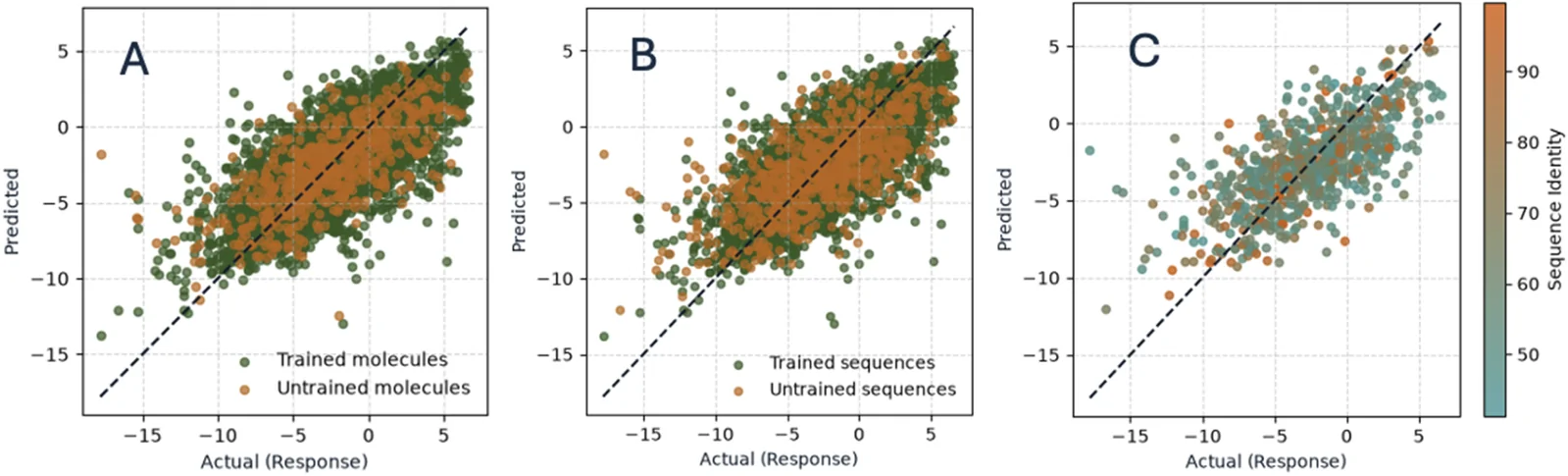
Assessment of model generalizability across molecular novelty and protein sequence similarity. The *x*-axis shows experimentally measured log_2_(*K*_m_) values, while the *y*-axis shows model-predicted log_2_(*K*_m_) values. (A) Predicted *versus* experimental values for test samples grouped according to whether the molecules were present in the training set. (B) Predicted *versus* experimental values for test samples grouped according to whether the protein sequences were identical to sequences in the training set. (C) Predicted *versus* experimental values for test proteins whose sequences were not identical to any sequence in the training set. Colors indicate the highest pairwise sequence identity between each test protein and the training-set proteins, calculated from MUSCLE-generated sequence alignments. Warmer and cooler colors represent higher and lower sequence identities, respectively. The diagonal line represents the ideal prediction (*y* = *x*).

A key finding of this study is the asymmetric contribution of protein and molecular representations to *K*_M_ prediction. While molecular features extracted sufficient information at relatively low dimensionality, protein-derived features benefited from higher-dimensional representations. This asymmetry likely reflects the greater complexity of protein sequence information involved in determining *K*_M_ property of protein-substrate pairs, including sequence-dependent structural and catalytic context. These observations highlight the importance of treating protein and substrate representations differently when designing machine learning models for enzyme kinetic prediction, rather than enforcing uniform feature dimensionality across inputs.

In addition to language model-based representations, we incorporated QM descriptors derived from electronic structure calculations to provide complementary physicochemical information. Specifically, the QM descriptors included HOMO energy, LUMO energy, chemical potential, chemical hardness, chemical softness, electrophilicity index, electron-donating power, and electron-accepting power. These descriptors characterize the electronic properties of molecules from different perspectives and are closely related to chemical reactivity. The HOMO and LUMO energies reflect the frontier molecular orbitals that govern a molecule's propensity to participate in electron–transfer interactions and other frontier-orbital-controlled reactions. Chemical potential describes the overall tendency of a molecule to exchange electrons with its environment, whereas chemical hardness and softness quantify its resistance or susceptibility to changes in electron density. The electrophilicity index, electron-donating power, and electron-accepting power further characterize the ability of a molecule to accept or donate electron density during chemical interactions. Because enzyme-catalyzed reactions depend not only on substrate recognition but also on the intrinsic electronic properties governing chemical transformation, these QM descriptors provide complementary information beyond structural representations and may therefore improve the prediction of enzyme kinetic parameters. Although QM descriptors are rarely integrated alongside language model representations in current *K*_M_ prediction studies, our results show that their inclusion consistently improves model performance across all tested feature configurations. This finding supports the value of combining data-driven representations with physics-based descriptors and demonstrates the potential of this hybrid strategy for enhancing enzyme kinetic modeling.

From a methodological perspective, the use of an overlapped sliding-window strategy enabled effective encoding of long protein sequences that exceed the input length limitations of current PLMs. Together, these designs improve the robustness and generalizability of the proposed framework and make it suitable for diverse enzyme-substrate scenarios encountered in metabolic studies.

Despite these advances, several limitations remain. The current models do not explicitly capture variations in *K*_M_ arising from enzyme-substrate interactions or the detailed mechanisms underlying enzymatic catalysis. Consistent with this limitation, the QM descriptors used in this study were not intended to directly represent the mechanistic determinants of *K*_M_, but rather to provide complementary information about substrate electronic properties. Future work will explore descriptors that more directly characterize enzyme-substrate interactions, such as protein-ligand interaction fingerprints, as well as metabolism-related features, including site-of-metabolism (SOM) descriptors and more fine-grained QM-derived properties. In addition, extending the current framework to jointly predict *K*_M_ and *K*_cat_ through multi-task learning may further improve predictive performance by leveraging shared information between substrate recognition and catalytic turnover.

Beyond predicting enzyme kinetic parameters, our model has the potential for several practical applications in enzymatic engineering and drug metabolism studies. For example, it could assist in identifying the most likely metabolic pathways of drug candidates by ranking enzymes according to their predicted catalytic activities toward a given compound. In addition, the framework could be further extended to investigate the effects of amino acid mutations on enzyme kinetics. By incorporating sequence variants, the model may help identify mutations that enhance catalytic activity or alter substrate specificity, providing a computational tool to support enzyme engineering.

## Conclusions

This study systematically implemented 4 PLMs and 2 MLMs for protein and substrate embedding extraction for downstream *K*_M_ prediction. We identified a feature configuration consisting of 70 protein-derived features from the last four layers of the ProtT5 PLM and 20 CLS-based molecular features from the MolFormer MLM as a well-balanced and effective representation for *K*_M_ prediction. Further increases in feature dimensionality resulted in only marginal performance gains. We also incorporated QM descriptors to further improve model performance, with the *R*^2^ reaching 0.537 compared to models without QM features under otherwise identical conditions. Together, this study provides a flexible and interpretable framework with strong potential to facilitate enzyme pathway identification, large-scale drug candidate screening, and sequence-based protein design, thereby supporting a broader range of applications in enzyme kinetics-driven modeling and drug development.

## Methods and materials

### Dataset collection and curation

The dataset was extracted from the BRENDA database and comprises 55 965 records of substrate-protein pair entries associated with experimentally measured *K*_M_ values describing enzyme-catalyzed reactions.^24^ These records include repeated substrate-protein combinations reported with different *K*_M_ measurements. For each entry, the dataset provides the substrate identifier (BRENDA compound number), protein EC number(s), UniProt ID(s), organism information, substrate name, and the corresponding *K*_M_ value. The dataset was retrieved in January 2025.

Substrate identifiers were manually mapped from BRENDA compound numbers to InChIKeys, which were subsequently matched to canonical SMILES strings obtained from PubChem.^25^ Protein sequences were retrieved based on UniProt IDs.^26^ As a result, substrates and proteins were consistently represented using canonical SMILES strings and amino acid sequences, respectively.

## Data cleaning and deduplication

As noted in the Introduction, a single protein sequence may be associated with multiple EC numbers. To avoid ambiguity and ensure consistency with machine learning input representations, protein uniqueness was defined at the amino acid sequence level rather than by EC number annotation.^14^ This sequence-based deduplication strategy prevents identical protein inputs from being associated with multiple labels, which could otherwise confound model training. After this filtering step, the dataset contained 8802 unique protein sequences and 4951 unique substrate SMILES.

To further ensure unambiguous training data, substrate-protein pairs were also deduplicated. After removing redundant entries, a total of 20 462 unique substrate-protein combinations were retained (SI Table 1). For duplicated substrate-protein combinations associated with multiple reported *K*_M_ values, the corresponding log_2_-transformed *K*_M_ values were averaged to generate a single representative record. This procedure reduces noise arising from experimental variability while preventing redundant inputs during model training. All *K*_M_ values were transformed to the log_2_ scale to improve data distribution.

### Language model input processing and embedding extraction

Four pretrained protein language models (PLMs), including ProtT5,^27^ ESM-2,^28^ ProtBERT,^29^ and Ankh,^30^ and two molecule language models (MLMs), ChemBERTa^31^ and MolFormer,^32^ were used to encode proteins and substrates, respectively.

For all language models, special tokens were enabled to explicitly indicate sequence boundaries and facilitate model recognition of input start and end positions. Protein sequences were processed individually rather than batched by length, eliminating the need for padding. For sequences exceeding the maximum input length supported by a given protein language model, an overlapped sliding-window strategy was applied.^33,34^ Each window covered 510 amino acids with a step size of 256 amino acids, ensuring partial overlap between adjacent windows to preserve local contextual continuity and minimize information loss at window boundaries. Sliding windows were generated sequentially along the full sequence length. For the final segment, the window length was maintained at 510 amino acids by extending upstream context when necessary (Fig. 2).

For ProtT5 and Ankh, amino acid-level embeddings were first generated, and sequence-level embeddings were derived from amino acid-level representations obtained from both the final transformer layer and the averaged representation of the last four layers, with equal contribution from each layer.^35,36^ For long sequences processed with sliding windows, amino acid embeddings appearing in multiple windows were first averaged across all occurrences before sequence-level aggregation, ensuring uniform weighting of each residue across the full-length sequence.^37,38^

Sequence-level embeddings for ESM-2 and ProtBERT were obtained following the similar procedure from the amino acid-level embeddings.^39^ In addition, both models provide a special sequence-level representation analogous to a classification token, along with an associated pooled embedding. Both classification-token embeddings and pooled embeddings were therefore extracted.^40^ For long sequences processed with sliding windows, classification-token and pooled embeddings were computed for each window and subsequently averaged to obtain a single sequence-level representation.

For the two molecule language models, ChemBERTa and MolFormer, no sliding-window processing was required. For each molecule, classification-token embeddings and pooled embeddings were directly extracted from the full input sequence.^41^

Given the high dimensionality of the raw embeddings and the potential risk of overfitting in downstream *K*_M_ prediction, PCA was applied to each set of model outputs to identify the most representative components.^42^ Only the top-ranked PCs were retained for subsequent model training, enabling more compact feature representations.

## QM feature

QM calculations were performed using the Gaussian 16 software.^43,44^ Geometry optimizations were carried out at the ωB97XD/6-31G* level of theory. Based on the optimized electronic structure, frontier molecular orbital (FMO) analysis was conducted to obtain the highest occupied molecular orbital (HOMO) and lowest unoccupied molecular orbital (LUMO) energies. We thus calculated the following term according to the equations:^45^IP ≈ −*E*_homo_EA ≈ −*E*_lumo_
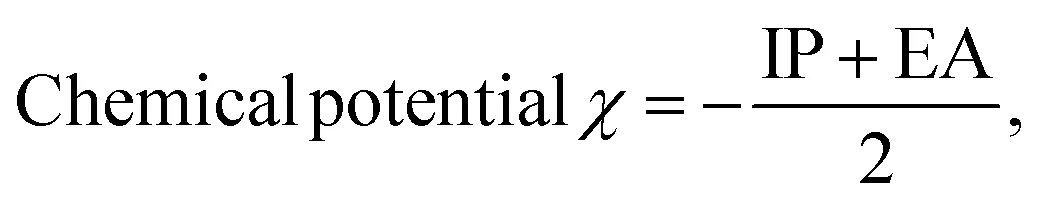

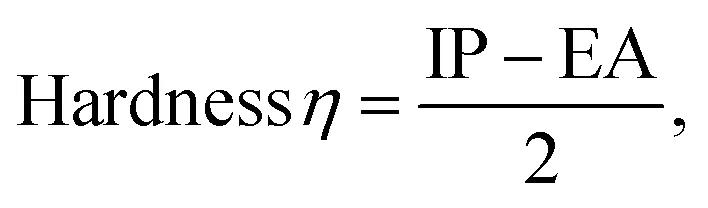

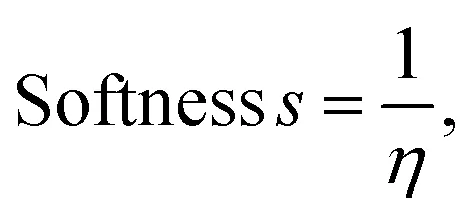

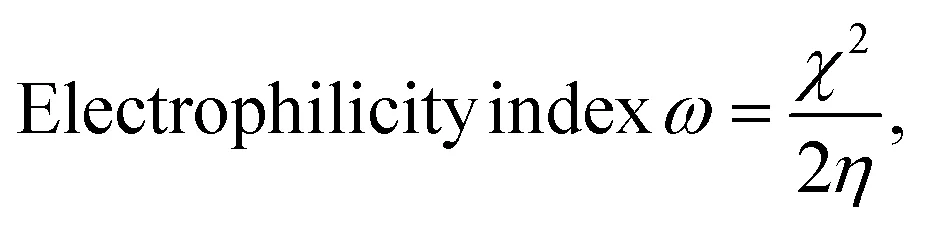

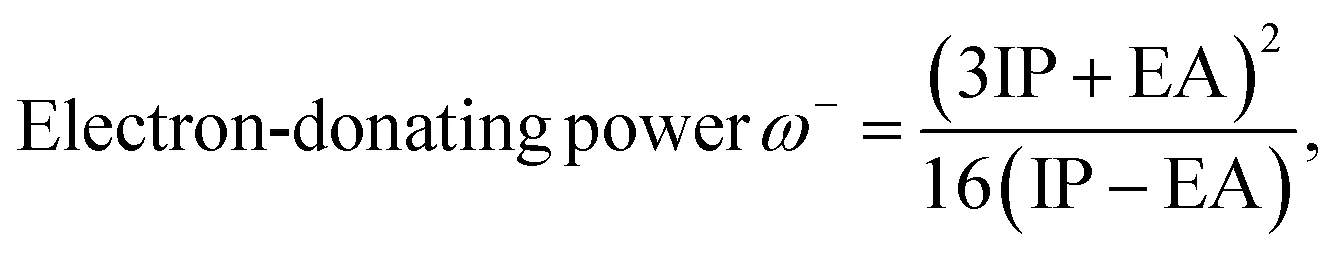

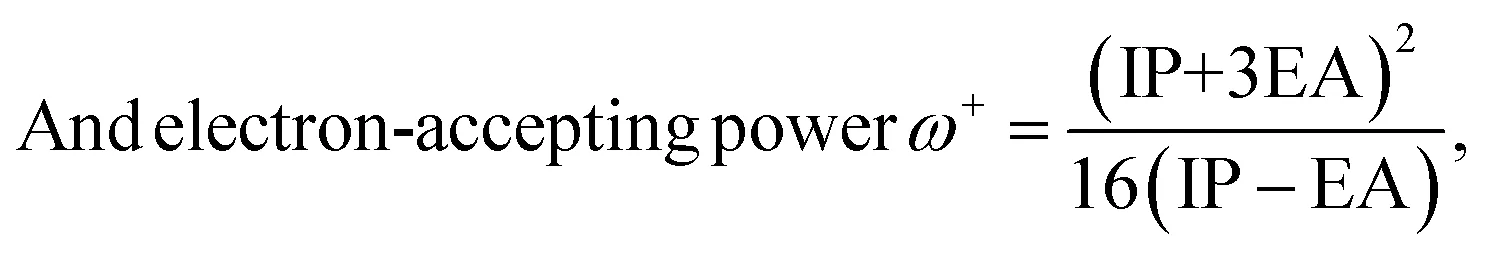


### LM2KM model

In our previous work on *K*_cat_ prediction, we constructed separate protein and substrate encoders and systematically evaluated multiple machine learning regression algorithms in MATLAB, among which ensemble bagged trees achieved the best performance.^18^ Following the same strategy, we conducted a similar model screening for *K*_M_ prediction, and ensemble bagged trees again outperformed other algorithms. The final ensemble bagged tree model was implemented in Python and was able to reproduce the performance obtained in MATLAB. Considering that the *K*_M_ data distribution is highly concentrated in the central range, with relatively sparse samples in both tails, additional weights were assigned to the top and bottom 10% of the data to improve model learning for underrepresented regions. The complete model implementation code is provided in the SI. The training and test sets were generated using fixed random seeds, and model training was fully reproducible by controlling random number initialization throughout the workflow.

## Evaluation metrics

Model performance was evaluated using the coefficient of determination (*R*^2^) and the root mean squared error (RMSE), calculated as follows:^46–48^
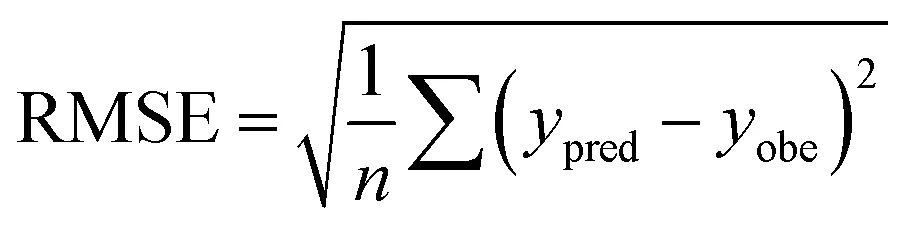
where *n* denotes the number of samples, *y*_obe_ represents the observed log_2_-transformed *K*_M_ values, and *y*_pred_ denotes the corresponding model predictions.

The coefficient of determination (*R*^2^) was computed as:
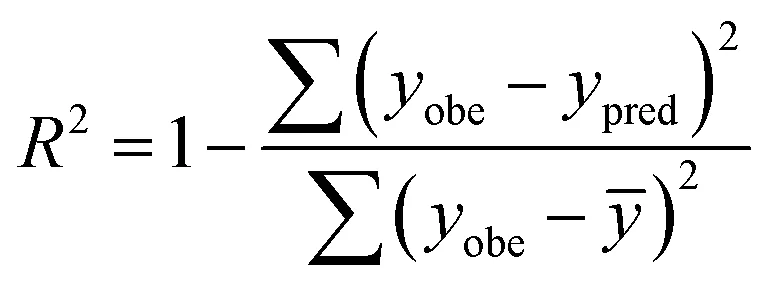
where *y*_obe_ and *y*_pred_ represents the same with previous, and *ȳ* is the mean of the observed log_2_-transformed *K*_M_ values in the evaluated dataset.

## Conflicts of interest

There are no conflicts to declare.

## Supplementary Material

DD-OLF-D6DD00139D-s001

## Data Availability

All datasets (PKL format) and source code (Jupyter Notebook/IPYNB format) generated and analyzed during this study are provided as supplementary information (SI) and are publicly available on Zenodo (DOI: https://doi.org/10.5281/zenodo.23020002). Supplementary information is available. See DOI: https://doi.org/10.1039/d6dd00139d. SI Fig: SI Fig. S1 shows the performance of PLM–MLM combinations using the top 20 principal components (PCs) from each model. SI Fig. S2 shows the performance of PLM–MLM combinations using the top 60 PCs from each model. SI Table: SI Table 1, supplementary_table_1.pkl, contains the dataset used for model development, comprising 20 462 unique substrate-protein combinations with corresponding (*K*_M_) values. Supplementary source code: supplementary_code_model_training.ipynb contains the source code for model development and training; supplementary_code_model_training_with-QM.ipynb contains the source code for model development and training with QM features, including comparisons between models with and without QM features. Supplementary descriptor datasets: supplementary_embeddings_summary.pkl contains the embedding representations of the entries in SI Table 1 generated using the PLMs and MLMs; supplementary_with-qm-features.pkl contains the subset used for model development with quantum mechanical (QM) features for 20 002 records.
