# Supplementary material for: LM2KM: feature extraction from pretrained protein and molecular language models combined with quantum mechanics (QM) features for KM predictions
Source: Digit Discov. 2026 Oct 20. Online ahead of print. doi: 10.1039/d6dd00139d (PMC13725812; doi:10.1039/d6dd00139d)
Supplement: Supplementary file 1 [file DD-OLF-D6DD00139D-s001.pdf]

Supplementary Information (SI) for Digital Discovery.
This journal is © The Royal Society of Chemistry

LM2KM: Feature extraction from pretrained protein and molecular language models combining with quantum mechanics (QM) features for K_M predictions

[Supplementary Figures]

Jingchen Zhai,[1] Lianjin Cai,[1] Taoyu Niu,[1] Lei Xie [2,3]*, Junmei Wang [1]*

1. Department of Pharmaceutical Sciences and Computational Chemical Genomics Screening Center, School of Pharmacy, University of Pittsburgh, Pittsburgh, Pennsylvania 15261, United States.

2. Department of Computer Science, Hunter College, The City University of New York, New York, NY, USA

3. Helen & Robert Appel Alzheimer's Disease Research Institute, Feil Family Brain & Mind Research Institute, Weill Cornell Medicine, Cornell University, New York, NY, USA

* Corresponding Authors:

Junmei Wang: juw79@pitt.edu,

Lei Xie: lxie@iscb.org

Key Words:

K_M prediction, drug metabolism prediction, machine learning, protein language model (PLM), molecular language model (MLM), embedding extraction

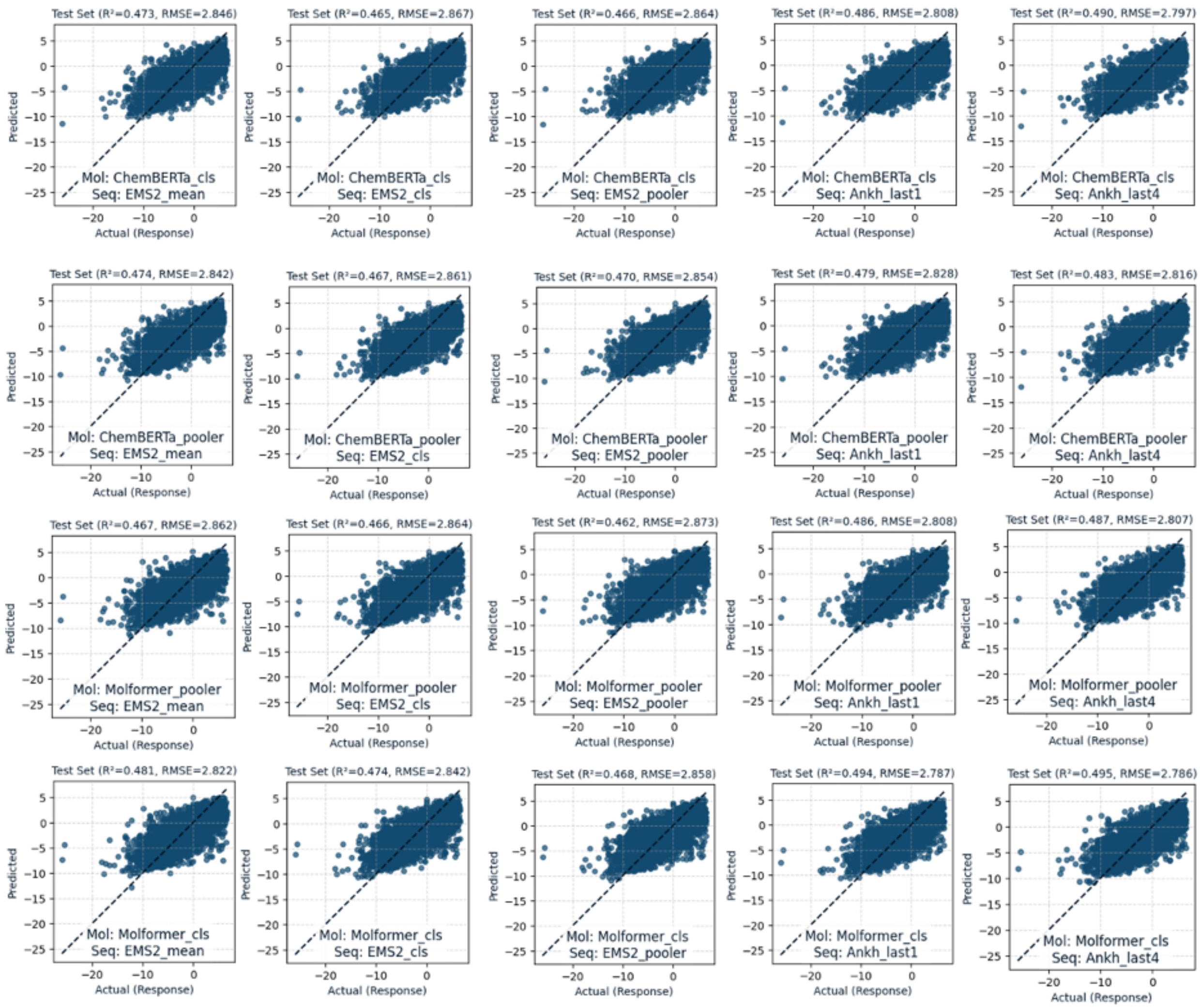

Supplementary Figure S1. PLM and MLM combinations applying 20 top PCs of each model.

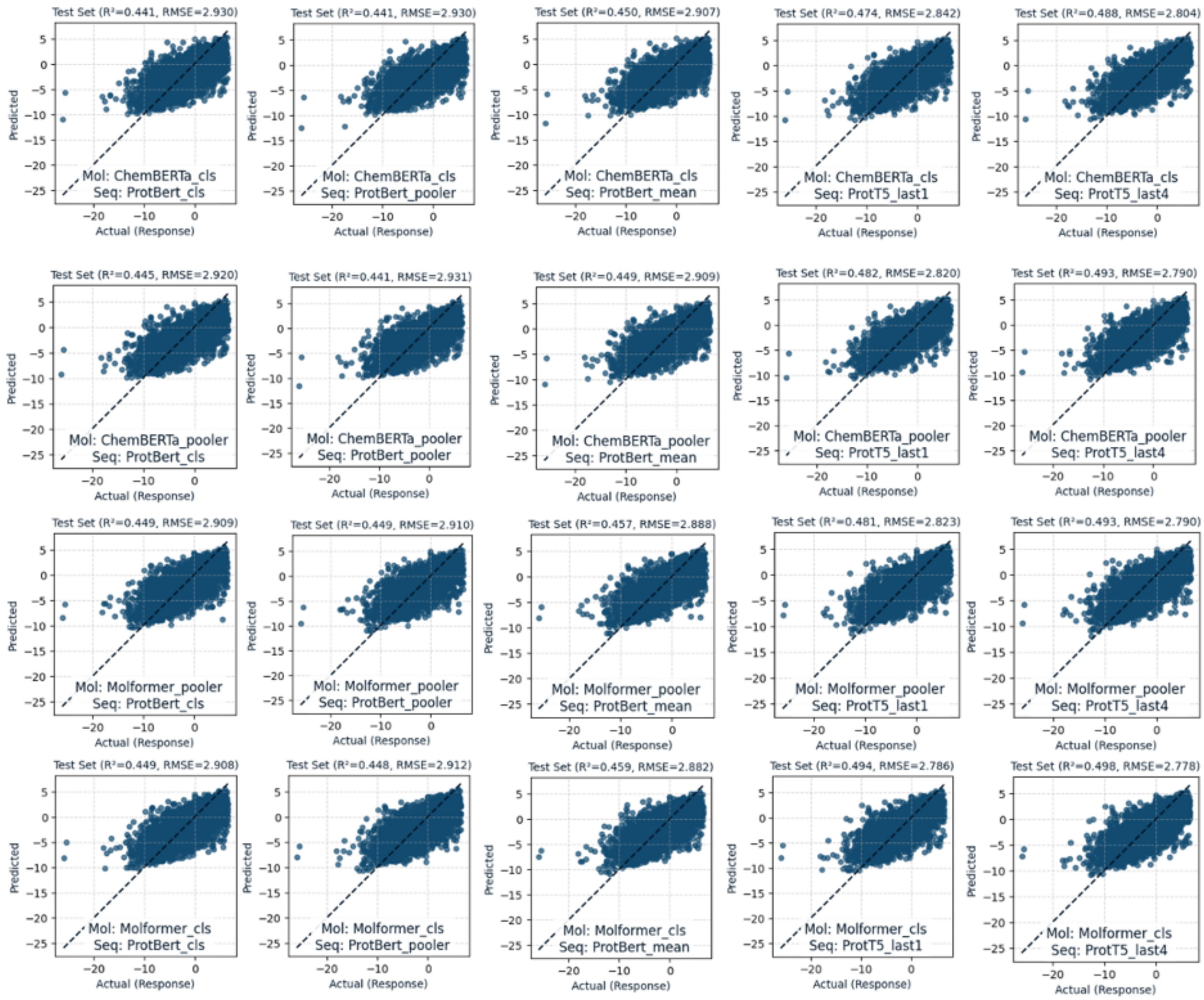

Supplementary Figure S1 (continued). PLM and MLM combinations using 20 top PCs of each model.

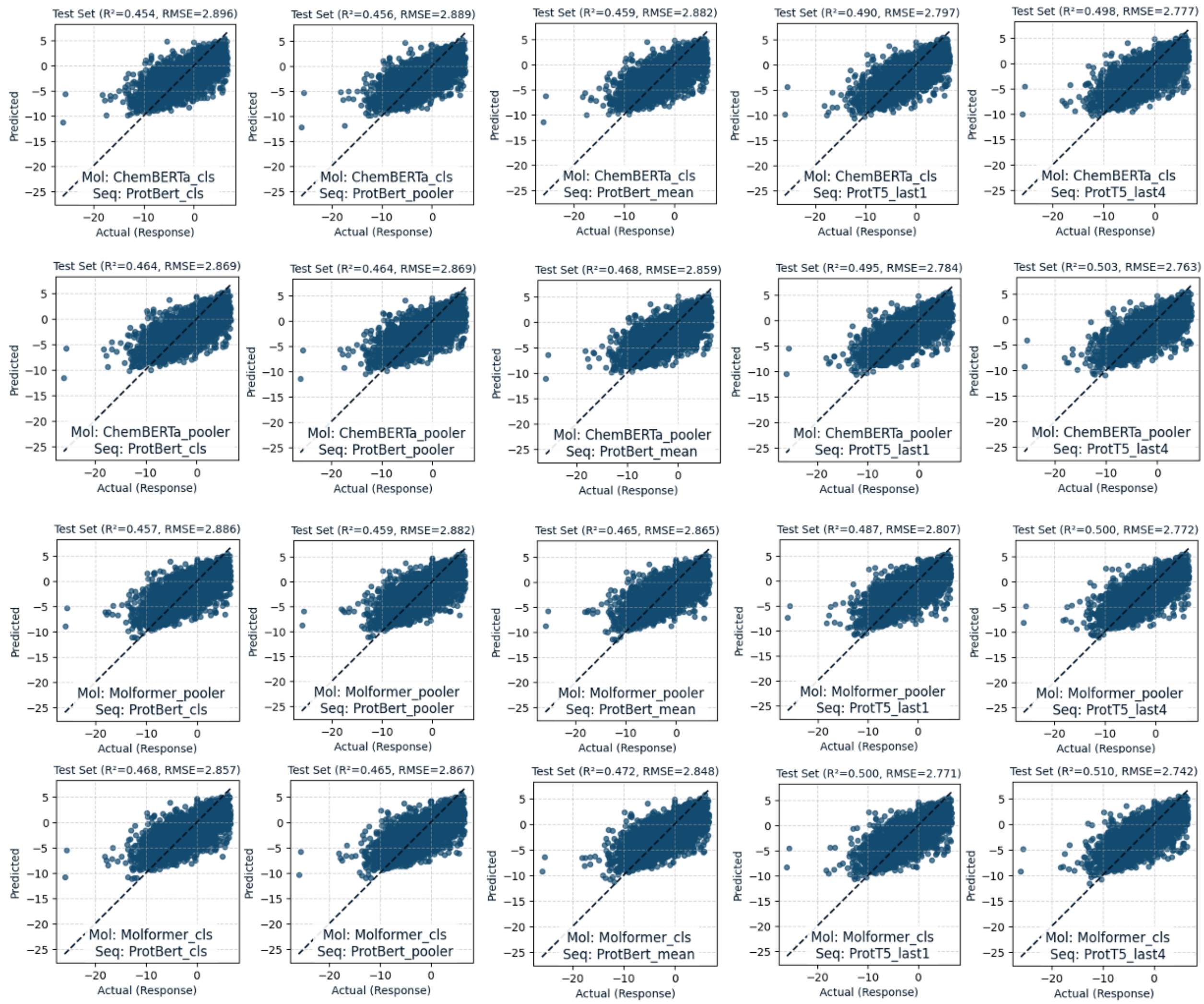

Supplementary Figure S2. PLM and MLM combinations applying 60 top PCs of each model.

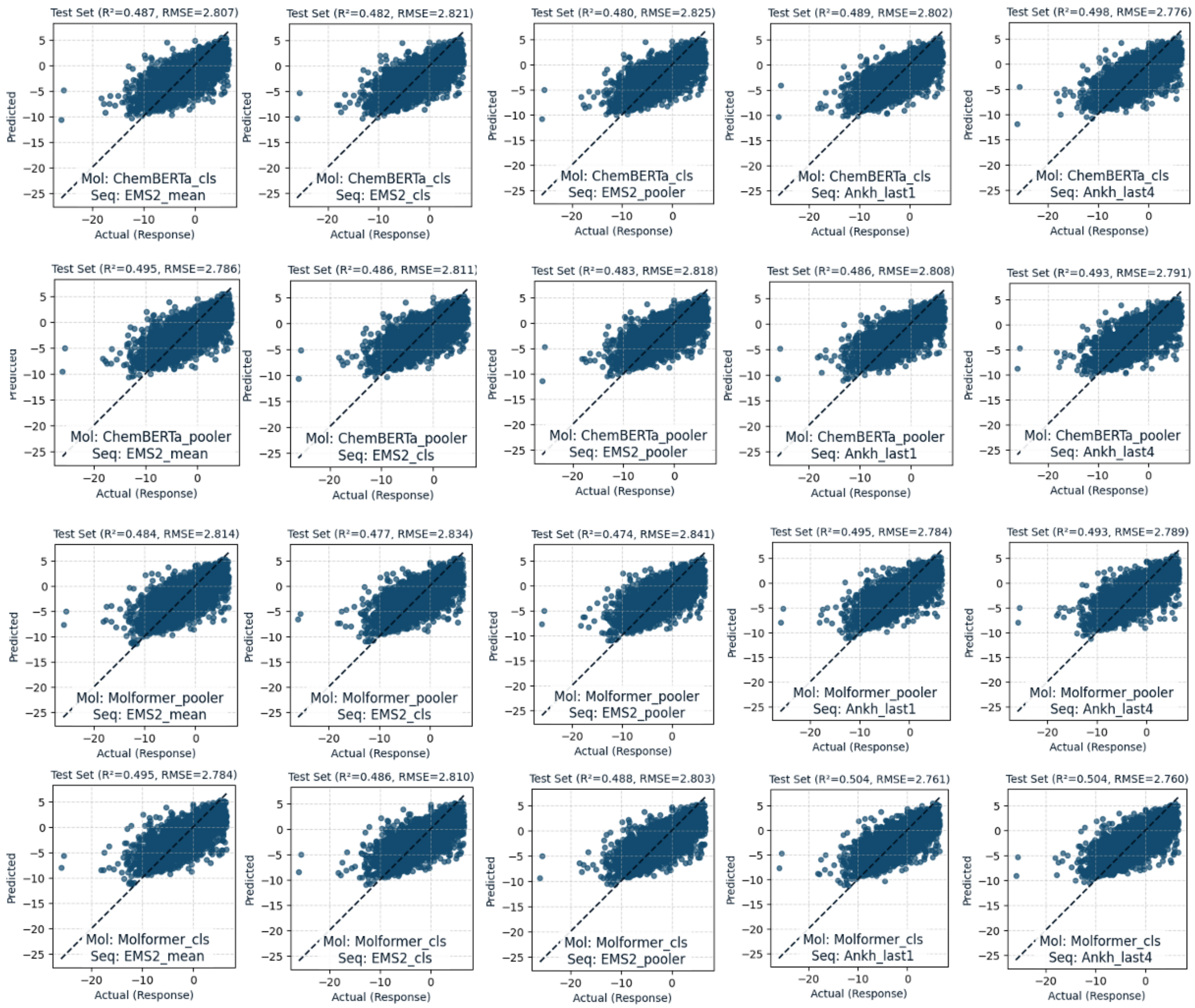

Supplementary Figure S2 (continued). PLM and MLM combinations using 60 top PCs of each model.
